# Risk factors in acquiring multidrug-resistant *Klebsiella pneumoniae* infections in a hospital setting in Saudi Arabia

**DOI:** 10.1038/s41598-023-38871-7

**Published:** 2023-07-19

**Authors:** Mutasim E. Ibrahim

**Affiliations:** Department of Basic Medical Sciences (Microbiology Unit), College of Medicine, University of Bisha, P. O. Box 731, Bisha, 67614 Saudi Arabia

**Keywords:** Diseases, Health care, Medical research, Risk factors

## Abstract

Over the last decades, the prevalence of multidrug-resistant (MDR) *Klebsiella pneumoniae* in clinical settings has increased progressively. This study determined the prevalence and risk factors associated with MDR *K. pneumoniae* infection among hospitalized patients in a referral hospital located in southern Saudi Arabia. A prospective cross-sectional study was conducted in King Abdullah Hospital from April 2021 to March 2022. *K. pneumoniae* (n = 211) bacteria were recovered from clinical samples of adult patients and examined for antibiotic susceptibility. Univariate and multivariate logistic regressions were applied to determine the factors associated with MDR *K. pneumoniae* infection. MDR *K. pneumoniae* strains was found in 66.8% (142/211) of the patients. Among MDR *K. pneumoniae*, the highest resistance rate was determined for ampicillin (100%), cefuroxime (97.9%), ceftriaxone (94.3%), and aztreonam (92.2%). The lowest resistance rate was determined for colistin (16.3%), and tigecycline (6.4%). Further, the patients’ gender, age group, intensive care unit (ICU) admission, invasive medical devices, and chronic illness were found to be significantly associated with MDR *K. pneumoniae* infection. The independent risk factors associated with MDR *K. pneumoniae* infection were the male gender (adjusted odds ratio [AOR] 2.107, 95% confidence interval CI 1.125‒3.945, *p* = 0.02), patients ≥ 65 years of age (AOR 1.905; CI 1.003‒3.616, p = 0.049), ICU admission (AOR 1.963; CI 1.033‒3.732, p = 0.04), diabetes (AOR 1.95; CI 1.02‒3.727, p = 0.043) and chronic obstructive pulmonary disease (AOR 7.172; CI 1.557‒33.032, p = 0.011). The study offered a vision of MDR *K. pneumoniae* infection in our setting and provided essential indications for further studies that may lead to the prevention and reduction of MDR bacteria.

## Introduction

*Klebsiella pneumoniae* (*K. pneumoniae*) is a gram-negative bacteria that cause frequent types of nosocomial infections^1,2^. Worldwide prevalence of hospital-acquired infections was reported as 8.7%, and about 10% were due to *K. pneumoniae*^1^. *K. pneumoniae* carries genes that mediate resistance to multiple antimicrobial drugs and can transfer these genes to other gram-negative bacteria^2^. Moreover, multidrug-resistant (MDR) *K. pneumoniae* cause different types of infection, including lower respiratory tract infections, bacteremia, bloodstream infections, wound infections and urinary tract infections^3^. The burden associated with MDR *K. pneumoniae* infections includes failure of treatment strategy, extended hospital stay, increased medical costs and increased mortality and morbidity rate^3,4^.

Over the last decades, the prevalence of MDR *K. pneumoniae* in clinical settings has increased progressively^1,5^. This is evidenced by several studies in Saudi Arabia revealing high rates of multidrug-resistant pathogens in hospital settings^6–9^. To illustrate, a surveillance dataset from a multi-hospital healthcare system in Saudi Arabia showed heavier and more resistant contribution of gram-negative pathogens to device-associated, healthcare-associated infections in comparison to American hospitals^6^.

Although many studies in Saudi Arabia have evaluated the MDR patterns of gram-negative pathogens, data considering the risk factors of acquisition of MDR pattern is still limited^8,10–12^. For example, in a study conducted at King Saud Medical City, a premier health centre in Saudi Arabia, Al Mayahi found that the uncontrolled use of antibiotics, prolonged stay in the intensive care unit (ICU) and frequent use of different devices are the potential risk factors in developing colistin resistance^13^. However, there is still a knowledge gap regarding the factors associated with MDR patterns among bacterial pathogens in many local regions^8,10^. Therefore, the risk factors that contribute to acquiring infection with MDR bacterial pathogens must be investigated. Thus, it can be hypothesized that the acquisition of infections with MDR pathogens might be correlated to patients’ sex^11^, age group^14^, hospitalization status, underlying condition and ICU admission^12^. More importantly, detecting such risk factors can facilitate the utilization of antibiotics in clinical practice and provide useful information to guide decision-making and control implementation of programs^11^. Therefore, this study aimed to determine the prevalence and risk factors associated with MDR *K. pneumoniae* infection among hospitalized patients in a referral hospital in southern Saudi Arabia.

## Materials and methods

### Study setting and design

This prospective cross-sectional study was conducted in King Abdullah Hospital (KAH) over a period from April 2021 to March 2022. The KAH is located in Bisha, a large province in the Aseer region in southern Saudi Arabia. It is the only main referral hospital with different departments and specialties that serve large populations of Bisha, the surrounding suburbs and cities of the neighbouring provinces.

### Study population

The study population was composed of adult patients aged 22 years or above who were admitted to KAH. Patients at this age group were more susceptible for bacterial infections, having comorbidities, frequently hospitalized and exposed to antibiotic treatments. The study excluded paediatric population, outpatient, patients with incomplete clinical data or patients with two types of bacterial infections. The general and clinical information of the patients were obtained from their medical records without violating their identities. However, patients’ identities not revealed anywhere in the study.

### Sample collection and processing

There was no precalculated sample size carried out for the study. All *K. pneumoniae* isolates recovered from clinical samples received at the hospital laboratory for routine microbiological investigations of infectious agents during the study period were included in the study.

The clinical samples of patients were collected and processed as per standard laboratory procedures^15^. Each patient’s mid-stream urine sample was collected and placed into a sterile plastic container, inoculated immediately on MacConkey agar and blood agar plates using a disposable calibrated plastic loop (1.3 mm diameter, delivering 1 μL) and incubated at 37 °C overnight. Wound pus was taken as a swab, placed on a transport medium and processed immediately by culturing MacConkey agar and blood agar plates (Oxoid Co., Cheshire, England). Sputum and other body fluids were collected into sterile plastic containers and inoculated directly onto MacConkey agar plates. All cultured plates were incubated aerobically for 24 h at 37 °C and were examined for countable colonies.

The blood samples (10 ml from each patient) were collected under a septic condition, inoculated immediately into tryptic soya broth (Oxoid Co., Cheshire, England) and incubated aerobically at 37 °C and daily checked for turbidity, haemolysis and clot formation. Each bottle with any turbidity was sub-cultured onto blood agar and MacConkey agar plates and incubated aerobically overnight.

Only one non-duplicate *K. pneumoniae* isolated from the patient sample was included in the study.

### Identification of *K*. *pneumoniae*

Identification of *K. pneumoniae* isolates was first done using colony characteristics, gram reaction and conventional biochemical tests. The isolates were then confirmed as *K. pneumoniae* by VITEK II automatic identification system (bioMerieux, Marcy l’E’toile, France) using the card for gram-negative strains (ID-GNB).

### Susceptibility tests

The susceptibility of *K. pneumoniae* isolates were categorized into different antibiotic classes using VITEK II cards (AST-N292) (bioMérieux, Marcy l’Etoile, France) per the instructions of the manufacturer. The minimum inhibitory concentration (MIC) and breakpoints were determined following the policies of the Clinical and Laboratory Standards Institute (CLSI)^16^. The antibiotics that were tested include amikacin, amoxicillin/clavulanate, ampicillin, aztreonam, cefepime, ceftriaxone, cefuroxime, ciprofloxacin, colistin, gentamicin, imipenem, meropenem, piperacillin/tazobactam, tigecycline and trimethoprim/sulfamethoxazole. *K pneumoniae* ATCC 700,603 and *Escherichia coli* ATCC 25,922 were used as quality control strains. The findings of antibiotic susceptibility were reported as resistant or sensitive. *K. pneumoniae that* is resistant to one antibiotic into three or more different antibacterial were identified as MDR isolates^17^.

### Detection of risk factors

To detect the risk factors associated with MDR *K. pneumoniae* infection, the patients’ general and clinical information were obtained from their clinical records in the hospital. These include patient gender, age group, type of infection, duration of hospital admission, ICU admission, utilization of invasive devices and comorbidities (diabetes mellitus, hypertension, chronic kidney disease, heart disease, cardiovascular disease, stroke, chronic obstructive pulmonary disease and COVID-19). It has assumed that change of antibiotic prescribing and infection control measures in the hospital settings during COVID-19 pandemic might have effect on the occurrence of MDR bacteria^18^.

### Ethics clearance

The study protocol was reviewed and approved by the Research Ethics Local Committee at the College of Medicine, University of Bisha (UBCOM-RELOC/H-06-BH-087 (05/04); on April 12th, 2021), with a waiver of informed consent. Study data and clinical information were collected from electronic database of the hospital and managed in anonymous format without violating patients’ identities. All methods were carried out in accordance with relevant ethical guidelines and regulations.

### Statistical analysis

Data were encoded and analysed using IBM SPSS version 28.0 (SPSS Inc., Chicago, IL, USA). Descriptive statistics was performed to describe baseline characteristics and presented in number, frequency, percentage, mean and standard deviation (SD). Risk factors associated with MDR *K. pneumoniae* infections were determined based on univariate and multivariate logistic regressions. The Pearson chi-squared test was used to compare categorical data. Fisher’s exact test was used when at least one cell count in the contingency table of the expected frequencies was less than five. In univariate logistic regression, the associations for acquiring MDR *K. pneumoniae* infections were presented with an odds ratio (OR) with a 95% confidence interval (95% CI). Variables with p-values < 0.2 in the univariate model were included in the multivariate logistic regression analysis. The backward stepwise elimination method was used to obtain the final predictive factors independently associated with MDR *K. pneumoniae* infection. Hosmer–Lemeshow goodness-of-fit test was used to assess model fits. The results were interpreted as an odds ratio in a 95% confidence interval (95% CI). All results with p-value < 0.05 were considered statistically significant.

## Results

Table 1 shows the baseline characteristics of the study participants with *K. pneumoniae* infection. A total of 211 *K. pneumoniae* were recovered from adult patients (n = 211). The mean age of the patients was 63.98 years (SD 19.8). One hundred twenty-five (59.2%) were aged 65 years or older, and the remaining were below that (40.8%). *K. pneumoniae* isolates were obtained from clinical samples of urine (n = 85), sputum (n = 74), wound pus (n = 25), blood (n = 20) and tracheal aspirate (n = 7). One hundred and seven (50.7%) isolates were recovered from patients who were in the ICU and 104 (49.3%) from other wards. One hundred and forty two (67%) patients were being hospitalized for less than one week while 32.7% (n = 69) were hospitalized for more than one week. Half of the patients (50.7%; 107) were admitted to the ICU and 75.8% (n = 160) with chronic diseases (Table 1).

**Table 1:** Baseline and clinical characteristics of patients with *Klebsiella pneumoniae* infection.

Characteristic	Total number of *K. pneumoniae* (n = 211)	n (%) of MDR *K. pneumoniae*
Gender		
Man	127	94 (74)
Woman	84	47 (56)
Sample		
Urine	85	50 (58.8)
Sputum	74	60 (81.1)
Wound	25	14 (56)
Blood	20	13 (57.1)
Tracheal aspirate	7	4 (66.8)
Age group		
≥ 65 year	126	92 (73)
< 65 year	85	49 (57.6)
ICU admission		
Yes	107	81 (75.7)
No	104	60 (57.7)
Duration		
≥ 1 week	142	93 (65.5)
< 1 week	69	48 (69.5)
Mechanical device usage		
Yes	69	53 (76.8)
No	142	88 (62)
Ventilated		
Yes	47	34 (72.3)
No	164	107 (65.2
Chronic disease		
Yes	160	115 (71.9)
No	51	26 (51)
Type of chronic disease		
DM	108	77 (72.1)
HTN	110	81 (73.6)
CKD	32	24 (75)
Heart disease	26	17 (65.4)
Cardiovascular	5	4 (80)
Stroke	48	33 (68.8)
COPD	23	21 (91.3)
COVID-19	10	8 (80)

*ICU* intensive care unit, *DM* diabetes mellitus, *HTN* hypertension, *CKD* chronic kidney disease, *COPD* chronic obstructive pulmonary disease.

Of the 211 patients, 141 (66.8%) were infected with MDR *K. pneumoniae*. These isolates were more frequently in men (74%, 94); in patients over 65 years (64.5%; 91); from sputum samples (81.1%; 60); patients in the ICU (75.7%; 81); patients who utilized mechanical ventilation (76.8%; 53) and those with chronic diseases (71.9%; 115) (Table 1).

Antibiotic susceptibility of MDR and non-MDR *K. pneumoniae* are illustrated in Table 2. Significant differences in resistance rate were detected between MDR and non-MDR isolates to all tested antimicrobial agents. Among MDR *K. pneumoniae*, the highest level of resistance was determined for ampicillin (100%), cefuroxime (97.9%), ceftriaxone (94.3%) and aztreonam (92.2%). Moderate resistance rates were observed for gentamicin (73%), amikacin (71.6%) and meropenem (63.8%). Low resistance rates were determined for imipenem (46.8%), colistin (16.3%) and tigecycline (6.4%).

**Table 2:** Antibiotic resistance profile of MDR and non-MDR *Klebsiella pneumoniae* isolates.

Antibiotics	*Klebsiella pneumoniae* isolates			P value
	Overall (n = 211), n(%) of resistance	MDR (n = 141), n(%) of resistance	Non-MDR (n = 70), n(%) of resistance	
Amikacin	101 (47.9)	101 (71.6)	0 (0.0)	< 0.001
Amoxicillin/clavulanic acid	132 (62.6)	128 (90.8)	4 (5.7)	< 0.001
Ampicillin	187 (88.6)	141 (100)	46 (65.7)	< 0.001
Aztreonam	136 (64.5)	130 (92.2)	6 (8.6)	< 0.001
Cefepime	136 (64.5)	130 (92.2)	6 (8.6)	< 0.001
Ceftriaxone	141 (66.8)	133 (94.3)	8 (11.4)	< 0.001
Cefuroxime	156 (73.9)	138 (97.9)	18 (25.7)	< 0.001
Ciprofloxacin	129 (61.1)	124 (87.9)	5 (7.1)	< 0.001
Colistin	23 (10.9)	23 (16.3)	0 (0.0)	< 0.001
Gentamicin	104 (49.3)	103 (73)	1 (1.4)	< 0.001
Imipenem	66 (31.3)	66 (46.8)	0 (0.0)	< 0.001
Meropenem	91 (43.1)	90 (63.8)	1 (1.4)	< 0.001
Piperacillin/tazobactam	126 (59.7)	115 (81.6)	11 (15.7)	< 0.001
Tigecycline	9 (4.3)	9 (6.4)	0 (0.0)	0.031
Trimethoprim/sulfamethoxazole	157 (74.4)	133 (94.3)	24 (34.3)	< 0.001

Univariate unadjusted analyses revealed an association between MDR *K. pneumoniae* and several demographic and clinical variables. Many characteristics, including the patients’ gender, age group, ICU admission, utilization of invasive medical device, presence of chronic illness, diabetes mellitus (DM), hypertension and chronic obstructive pulmonary disease (COPD) were significantly associated with MDR *K. pneumoniae* infection. As illustrated in Table 3, men were two times more likely to acquire infection with MDR *K. pneumoniae* than women (odd ratio [OR] 2.242; 95% confidence interval CI 1.249‒4.027, *p* = 0.07). There were increased OR for MDR *K. pneumoniae* infection among patients aged 65 years or above (OR 1.988; 95% CI 1.11–3.561, *p* = 0.021) than those aged below 65 years old. Patients admitted to the ICU were two times more likely to have MDR *K. pneumoniae* than those in the other hospital wards (OR 2.285; 95% CI 1.268–4.116, *p* = 0.006). Moreover, an almost similar increase in OR for MDR *K. pneumoniae* infection was observed among patients subjected to mechanical devices than those without it (OR 2.033; 95% CI 1.057–3.908, *p* = 0.033). Moreover, patients with chronic diseases were more likely to acquire MDR *K. pneumoniae* infection than those without chronic diseases (OR 2.457; 95% CI 1.285–4.699, *p* = 0.007). However, there were increased odds of infection among patients with COPD, DM and hypertension. Other examined variables, such as period of hospitalization, type of clinical samples, usage of ventilation, chronic kidney disease (CKD), heart disease, cardiovascular diseases, stroke and ccoronavirus disease (COVID-19), were not significantly related to MDR *K. pneumoniae* acquisition. Detailed data are presented in Table 3.

**Table 3:** Potential risk factors for multidrug-resistant *Klebsiella pneumoniae* infection in adult patients in univariate analysis.

Characteristic	Un-adjusted odds ratio (% 95 CI)	P value
Sex		
Man	2.242 (1.249–4.027)	**0.070**
Woman	1	
Age group		
≥ 65 years	1.988 (1.11–3.561)	**0.021**
< 65 years	1	
ICU admission		
Yes	2.285 (1.268–4.116)	**0.006**
No	1	
Duration		
≥ 1 week	0.830 (0.447–1.541)	0.556
< 1 week	1	
Device		
Yes	2.033 (1.057–3.908)	**0.033**
No		
Sample		
Urine	1.071 (0.226–5.089)	0.931
Sputum	3.214 (0.645–16.016)	0.154
Wound	0.955 (0.176–5.186)	0.957
Blood	1.393 (0.24–8.067)	0.712
Tracheal aspirate	1	
Ventilated		
Yes	1.393 (0.681–2.849)	0.364
No	1	
Chronic disease		
Yes	2.457 (1.285–4.699)	**0.007**
No	1	
DM		
Yes	1.650 (0.926–2.939)	**0.088**
No	1	
HTN		
Yes	1.909 (1.068–3.412)	**0.028**
No	1	
CKD		
Yes	1.590 (0.675–3.747)	0.286
No	1	
Heart disease		
Yes	0.929 (0.392–2.205)	0.868
No	1	
Cardiovascular		
Yes	2.015 (0.221–18.370)	1.00
No	1	
Stroke		
Yes	1.120 (0.561–2.237)	0.747
No	1	
COPD		
Yes	5.950 (1.354–26.154)	**0.008**
No	11	
COVID-19		
Yes	2.045 (0.423–9.897)	0.365
No	1	

*ICU* intensive care unit, *DM* diabetes mellitus, *HTN* hypertension, *CKD* chronic kidney disease, *COPD* chronic obstructive pulmonary disease.

Significant values are in bold.

In the multivariate analyses (Table 4), predictors of infections with MDR *K. pneumoniae* included being male, patients aged 65 years or older, ICU admission, DM and COPD.

**Table 4:** Results of multivariate logistic regression analysis showing factors associated with acquisition of infection with MDR *Klebsiella pneumoniae* strain.

Factor	Adjusted odds ratio (% 95 CI)	P value
Male patient	2.107 (1.125–3.945)	0.02
Patients of ≥ 65 years	1.905 (1.003–3.616)	0.049
ICU admission	1.963 (1.033–3.732)	0.040
Diabetes mellitus	1.95 (1.02–3.727)	0.043
Chronic obstructive pulmonary disease	7.172 (1.557–33.032)	0.011

## Discussion

The present study identified the common factors that increased the risk of acquiring infection with MDR *K. pneumoniae* among hospitalized patients. In this study, the prevalence of MDR pattern among *K. pneumoniae* was found to be at 66.8%. This is higher than the 16.9% reported in eastern Saudi Arabia from 2017 to 2018^11^ and the 23% reported in a study conducted in Riyadh, the capital of Saudi Arabia, from 2008 to 2016^6^. Furthermore, a study that estimated the resistance of gram-negative pathogens causing surgical site infections at a multi-hospital healthcare system in Saudi Arabia within 2007–2016 found that the proportion of MDR *K. pneumoniae* was 20.4%^19^. Worldwide studies documented the occurrence of MDR patterns among bacterial pathogens. For instance, a systematic review recorded data from 28 countries of six regions found that the pooled prevalence of nosocomial MDR *K. pneumoniae* was estimated at 32.8% (95% CI 23.6–43.6)^1^. The proportions of MDR *K pneumoniae* clinical isolates have been reported in Asian countries—for instance, 46.6% rate in Pakistan^20^ and 70% in Indian hospitals^21^. Notably, the percentage of MDR *K. pneumoniae* vary geographically and has become a significant concern around the globe. The differences in resistance rates among geographical regions could be attributed to the variations in the types of patients served, the policy of antibiotic use or the lack of hospital hygiene and infection-control measures^22,23^. However, the widely used antibiotics in hospital settings lead bacterial pathogens to develop acquired resistance to many antibiotic classes^4,21^. In addition, the horizontal transfer of mobile genetic elements carrying resistant determinants to several antimicrobial types is the major driver of resistance in gram-negative bacteria^24^. Today, more than 100 acquired resistance genes have been identified in *Klebsiella* strains, conferring the susceptibility of wide ranges of antimicrobial agents^2,25^. This corroborates the need to continue molecular epidemiology studies to assess the resistance genotypes and their diversity and distribution in each region^8^.

In the present study, MDR *K. pneumoniae* were commonly recovered from sputum samples, indicating that the respiratory system is the most common source of MDR *K. pneumoniae* strains in this clinical setting. Similarly, a study from India showed that MDR *K. pneumoniae* were commonly isolated from sputum samples^21^. On the contrary, a study conducted in eastern Saudi Arabia found that urine is a common source of MDR gram-negative bacteria, including *K. pneumoniae*^11^. Furthermore, a study from Thailand specified the urinary system is the most important site of infection-producing MDR-gram-negative bacteria^26^. This might be due to the differences in antibiotic use, environmental conditions and resistance patterns in each specific geographical region, population and healthcare facilities. Therefore, understanding the susceptibility patterns and distribution of MDR bacteria in a given hospital setting is important in selecting empirical therapy that leads to the desired outcome for the patients^4,7^.

In the present study, MDR *K. pneumoniae* revealed high resistance to ampicillin, cefuroxime, ceftriaxone, and aztreonam. This finding is similar to those reported from the eastern region of the country where the proportions of resistance were more than 70% for ampicillin, amoxicillin/clavulanic acid, amoxicillin/clavulanic acid, ceftriaxone and cefuroxime^11^. In addition, a study indicated that *K. pneumoniae* has become resistant to third-generation cephalosporins and carbapenems, which led to narrowing therapeutic choices for MDR *K. pneumoniae* infection^2^. The high resistance rates could be explained by these antibiotics being the most prescribed therapeutic agents in clinical settings. It is well backed up by data that frequent use of antibiotics is a significant risk factor associated with the development of bacterial resistance^7,10,27^. For instance, the overuse of meropenem and piperacillin-tazobactam antibiotics has been reported in adult ICUs in Saudi Arabia^25^. This necessitates controlling inappropriate prescription of antibiotics, including antibiotic selection, dosing, or duration and align them with evidence-based recommendations for diagnosis and management in clinical settings^28–30^. More importantly, establishing a local antibiogram database will guide the treatment strategies for bacterial pathogens^7^.

In the univariate analysis, the study identified several factors correlated with MDR *K. pneumoniae* infections. The result showed that men were more likely to have infections with than women. Consistently, the number of MDR *K. pneumoniae* was higher in male (64.4%) than in female patients (35.7%) found in a study carried out in China^4^. Various findings in scientific literature documented the association between patients’ gender and MDR pathogens. In a study determining the prevalence of MDR gram-negative bacteria and its associated risk factors from patients in the Security Forces Hospital in Dammam, Saudi Arabia, Al Hamdan et al.^11^ found that the resistance was not significantly different between the male and female patients (*p* = 0.084). In the United States, a study found no significant associations between the gender of patients and the presence of MDR among extended-spectrum *b-*lactamase–producing *Escherichia coli* and *Klebsiella* species^31^. Similarly, a study in Iran reported no significant associations (*p* = 0.63) between MDR *K. pneumoniae* and being male or female^20^. On the contrary, the female sex was found to be independently associated with the acquisition of MDR pathogens in a study carried out in Ghana^14^. The higher rate of MDR *K. pneumoniae* found among men in this study may be attributed to the fact that male patients have more complications and serious conditions that need extensive therapy and invasive and non-invasive procedures. However, the frequent use of specific antibiotics provides a selective pressure for MDR in bacteria pathogens^4^. Therefore, further longitudinal studies are needed to understand the gender differences in the prevalence of infections caused by MDR *K. pneumoniae*.

In this study, patients  ≥ 65 years were about two times to acquire MDR *K. pneumoniae* infections than those below 65 years old. This is almost consistent with the finding of Gnimatin et al. who found that patients 60 years old and above (crude OR 1.41; 95% CI 1.05–1.89; p-value = 0.023) had 1.41 times more odds of developing multidrug-resistant organisms infections^14^. A recent study in Saudi Arabia reported that patients aged 70 to 79 years and more than 90 years are at risk of MDR-gram-negative bacterial infection^11^. A possible explanation is that elderly patients are more frequently hospitalized, likely to be immune-compromised and under frequent mechanical procedures and long-term antibiotic therapy. In addition, a previous study found that elderly patients are at a high risk of nosocomial infections due to a higher disease prevalence in this population, including neurological disorders, diabetes and cardiovascular diseases^4^.

The present study showed that ICU admission and exposure to invasive devices were risk indicators in acquiring infections with MDR *K. pneumoniae* strains, which is in agreement with several studies^3,4,8,32^. Furthermore, the increased risk of acquiring infections with MDR bacteria in the ICU is associated with the severity of the patient’s illness and underlying conditions, length of exposure to invasive procedures, extensive antibiotic use and increased patient contact with healthcare personnel and length of stay in the ICU^4,7,32^.

Furthermore, chronic diseases were predictors of MDR *K. pneumoniae* infections. However, patients with COPD were about six times more likely to have MDR *K. pneumoniae* infections. In addition, patients with hypertension have a high risk (OR = 1.909) of acquiring MDR pathogens. This is attributed to patients with chronic illness requiring a prolonged hospital stay, extensive antibiotic therapy and instrumentation such as prolonged bladder catheterization and mechanical ventilation^13^.

After adjusting for other confounding factors, the multivariate analysis resulted in five independent factors inclusive of male patients, elderly patients, COPD, DM and ICU admission associated with MDR *K. pneumoniae* infection in our setting. A previous study in Riyadh found that DM, hypertension, renal and heart diseases were common comorbidities among patients with colistin-resistant pathogens^13^. Patients with underlying diseases and serious conditions staying longer in hospital and usually under frequent use of devices, invasive procedures and broad spectrum of antibiotics might facilitate acquisition MDR pathogens^8,33^.

ICU admission has been documented as an independent risk factor for the acquisition of MDR *K. pneumoniae* by many authors^7,32,34^. It was suggested that ICU patients are at an increased risk of acquiring infections with MDR bacteria and most of which are associated with the use of invasive devices such as indwelling catheters and central venous catheters^4,7,31,35^. A previous study in Saudi Arabia suggested that increasing resistance rates in the ICU and surgical wards may be parallel with higher usage of antimicrobial drugs. Other factors, such as using other medications or cross-transmission, may play an important role in circulating these organisms in hospital wards^34^. Therefore, epidemiological studies are necessary to deliver important information about pathogen characteristics, their resistance, type of patients infected with and treatment outcomes and could be useful in the development and implementation of control policies in the ICU^7^. Moreover, practice of hygiene among healthcare providers is essential to reduce the circulation of MDR pathogens in the hospital settings^14^.

### Limitations

The present study has several limitations. First, the relatively small sample size of this cross-sectional study might restrict the study findings. Second, the study did not examine many possible risk factors including previous antibiotic uptake and clinical history. Therefore, analysis of more isolates against diverse factors would provide a more generalized database. Third, the study did not consider all the patients with *K. pneumoniae* infections in the hospital, but only those who collected their clinical samples for routine laboratory investigations of diseases and yielded positive culture results for *K. pneumoniae*. To generalize these findings, further studies that targeted all patients admitted with suspected *K. pneumoniae* infection are essential.

## Conclusions

The study reported increased MDR patterns among *K. pneumoniae* and high resistance rates against the tested antibiotics. The high resistance rate highlights the need to strengthen hospital-based antibiotic stewardship programs and infection-control measures to decrease the spread of MDR bacteria. Moreover, awareness of the susceptibility patterns of pathogens within a given local setting is important to select suitable antimicrobial therapy.

The study demonstrated the significance of identifying risk factors in acquiring multidrug-resistant bacteria to design and implement strategies to prevent further increase in MDR pathogens. Determining the risk factors in developing MDR bacterial infections provided essential information to combat infection caused by such pathogens and can preserve alternative antimicrobial agents, thereby reducing the dependence on carbapenems. This study has offered a vision of MDR *K. pneumoniae* infection in our hospital setting and provided essential indications for further studies that may serve as guide in the prevention and reduction of MDR bacteria.

## Data Availability

The data generated or analyzed during the study are included in this manuscript. However, the dataset is available upon reasonable request from the corresponding author.
